# Author Correction: Float-stacked graphene–PMMA laminate

**DOI:** 10.1038/s41467-025-65418-3

**Published:** 2025-10-24

**Authors:** Seung-Il Kim, Ji-Yun Moon, Seok-Ki Hyeong, Soheil Ghods, Jin-Su Kim, Jun-Hui Choi, Dong Seop Park, Sukang Bae, Sung Ho Cho, Seoung-Ki Lee, Jae-Hyun Lee

**Affiliations:** 1 Department of Energy Systems Research, Ajou University, Suwon, 16499 Korea; 2 Department of Materials Science and Engineering, Ajou University, Suwon, 16499 Korea; 3 Mechanical Engineering & Materials Science, Washington University in St. Louis, Saint Louis, MO 63105 USA; 4 Functional Composite Materials Research Centre, Institute of Advanced Composite Materials, Korea Institute of Science and Technology, Wanju, 55324 Korea; 5 A Development Team, Samsung Display, Asan, 31454 Korea; 6 Department of Materials Science and Engineering, Pusan National University, Busan, 46241 Korea

**Keywords:** Two-dimensional materials, Mechanical and structural properties and devices, Composites

Correction to: *Nature Communications* 10.1038/s41467-024-46502-6, published online 11 March 2025

In the version of the article initially published, some of the scale bars were mislabelled. In the Fig. 1 caption, the description now reading “Scale bar: 60 μm and 5 μm,” “60 μm” replaces “100 μm.” In the Supplementary Fig. 5 caption, “Scale bars: 50 µm, 47.6 µm, 50 µm, 35.6 µm, 35.6 µm, and 36.4 µm, respectively” replaces the original “Scale bar is 50 µm.” The analyses in this paper were based on the correct values of the scale bars.

In the “Reinforcing effect of the well-aligned graphene layers in the GPL” section, in the text now reading “…we prepared three different samples with 25 layers of GPM (A4, 1500 rpm),” “1500 rpm” replaces the original “500 rpm,” while in the following sentence now reading “The volume fraction of GPL with 25 layers of GPM (A4, 1500 rpm) is 0.190%,” 1500 rpm and 0.190% replace the original values 500 rpm and 0.098%.

There was also an error in the Methods “Characterization” section, where in the text now reading “Raman mapping was carried out on an area of 35 μm × 35 μm in 0.5 μm steps,” “35 μm × 35 μm” replaces the original “50 μm × 50 μm.” The errors have been corrected in the HTML and PDF versions of the article.

