# Supplementary material for: Float-stacked graphene–PMMA laminate
Source: Nat Commun. 2024 Mar 11;15:2172. doi: 10.1038/s41467-024-46502-6 (PMC10928174; doi:10.1038/s41467-024-46502-6)
Supplement: Supplementary file 1 — Supplementary Information [file 41467_2024_46502_MOESM1_ESM.pdf]

Supplementary Information for

Float-stacked graphene–PMMA laminate

Seung-Il Kim, Ji-Yun Moon, Seok-Ki Hyeong, Soheil Ghods, Jin-Su Kim, Jun-Hui Choi, Dong Seop Park, Sukang Bae, Sung Ho Cho, Seoung-Ki Lee, Jae-Hyun Lee

*Corresponding author: S.H.C. (sungho19.cho@samsung.com) S.-K.L. (ifriend@pusan.ac.kr) and J.-H.L. (jaehyunlee@ajou.ac.kr).

This Supplementary Information includes:

Supplementary Discussion

Supplementary Figures 1 to 16

Supplementary Tables 1 to 3

Supplementary References 1 to 19

Supplementary Discussion

1. Adhesion force of the GPM–water meniscus

We compared graphene-water and graphene–PMMA interfacial energies to confirm the membrane tension. The fracture energy (G) physically applied to the contacted interfaces to separate from each other can be expressed as the work of adhesion (W_A) by the following relation:[1, 2]

$$G = W_A \times \phi \tag{1}$$

where, ϕ is a viscoelastic term that depends on temperature and rate. According to Andrews et al., W_A is smaller than G, but it can have a large impact on practical adhesion (G)[3]. Therefore, by using the Young–Dupre equation and the Owens–Wendt model, we calculated and compared the work of adhesion between graphene–water and graphene–PMMA interface.

1.1. The Young–Dupre equation

According to the Dupre equation, the work of adhesion (W_{sl}) at the interface between solid and liquid is defined by the following equation:

$$W_{sl} = \gamma_s + \gamma_l - \gamma_{sl} \tag{2}$$

where, γ_{sl}, γ_s, and, γ_l are the surface energy of the soild–liquid, solid–vapor, and liquid–vapor interfaces, respectively. Combined with the Young–Laplace equation,

$$\cos\theta = \frac{\gamma_s - \gamma_{sl}}{\gamma_l} \tag{3}$$

The work of adhesion at the interface between solid and liquid (W_{sl}) can be expressed by the Young–Dupre equation:[4, 5]

$$W_{sl} = \gamma_l(1 + \cos\theta) \tag{4}$$

where, θ is the contact angle of the liquid droplet.

According to the surface energy of water, 72.80 mJ m^{-2}, and the contact angle of graphene of $(42 \pm 3)°$, the W_{sl} of the graphene–water interface was calculated to be 126.90 mJ m^{-2} [6].

1.2. The Owens–Wendt (extended Fowkes) model

The surface energy of the graphene–PMMA interface was calculated using the Owens–Wendt (extended Fowkes) model. The work of adhesion between solid and liquid can be dissociated into dispersive and polar components of two phases, according to the following equation:[7, 8]

$$W_{sl} = 2(\sqrt{\gamma_s^d \gamma_l^d} + \sqrt{\gamma_s^p \gamma_l^p}) \tag{5}$$

where, γ_s^d and γ_l^d are the dispersive components, and γ_s^p and γ_l^p are the polar components. The Owens–Wendt (extended Fowkes) model is based on the Young and Good equations, and the Good equation is as follows:

$$\gamma_{sl} = \gamma_s + \gamma_l - 2(\sqrt{\gamma_s^d \gamma_l^d} + \sqrt{\gamma_s^p \gamma_l^p}) \tag{6}$$

Combining Eq. (6) with Young's equation, the Owens–Wendt (extended Fowkes) model is expressed as follows:

$$\gamma_l(1 + \cos\theta) = 2(\sqrt{\gamma_s^d \gamma_l^d} + \sqrt{\gamma_s^p \gamma_l^p}) \tag{7}$$

or,

$$\frac{\gamma_l(1 + \cos\theta)}{2\sqrt{\gamma_l^d}} = \sqrt{\gamma_s^p}\frac{\sqrt{\gamma_l^p}}{\sqrt{\gamma_l^d}} + \sqrt{\gamma_s^d} \tag{8}$$

When the left side of Eq. (8) is y, $\frac{\sqrt{\gamma_l^p}}{\sqrt{\gamma_l^d}}$ is x, a linear fitting can be obtained, where γ_s^p is the square of the slope, and γ_s^d is determined as the square of the y-intercept.

To calculate the γ_s^p and γ_s^d of a solid, at least two liquid polar and dispersive components are required[6, 9]. (Here, we used the contact angle of PMMA with pure H_2O and CH_2I_2 ($\theta_{H_2O,PMMA} = (69 \pm 1)°$, $\theta_{CH_2I_2,PMMA} = (48 \pm 1)°$, $\theta_{H_2O,Graphene} = (42 \pm 3)°$, $\theta_{CH_2I_2,Graphene} = (31.7 \pm 1)°$). Based on that, we can calculate the polar and dispersive components of PMMA and graphene. The Owens–Wendt model is appropriate to calculate the surface energy of the solid–solid interfaces, as well as the solid–liquid interfaces. From Eq. (5), the work of adhesion of the graphene–PMMA interface was calculated to be 101.45 mJ m^{-2}.

Compared with the above calculation results, the work of adhesion of the graphene–water interface (126.90 mJ m^{-2}) is higher than that of the graphene–PMMA interface (101.45 mJ m^{-2}). Practical adhesion acting along the meniscus at the graphene–water interface is used as a sufficiently strong fracture energy for the graphene–PMMA interface in contrast, and the rolling GPM is subjected to tension to continuously separate from the stacked GPM.

2. *Mechanical model of the GPL*

2.1. *The Halpin–Tsai model*

The Halpin–Tsai model can be universally applied to parallel orientation aligned nanoplate reinforced nanocomposites[10]. The Young's modulus of the longitudinal direction is defined as follows:

$$E_{\parallel} = \left(\frac{1 + 2a\eta_{\parallel}V_f}{1 - \eta_{\parallel}V_f}\right)E_m \tag{9}$$

where, a is the aspect ratio (width/thickness) of the filler, V_f is the volume fraction of filler, E_m is the modulus of matrix, and $\eta_{\parallel}$ is defined as follows:

$$\eta_{\parallel} = \frac{\frac{E_f}{E_m} - 1}{\frac{E_f}{E_m} + 2a} \tag{10}$$

As $a \rightarrow \infty$, Eq. (11) reduces to the parallel model of the rule of mixtures.

2.2.Rule of mixtures

The rule of mixtures assuming good and void free stress transfer provides a simple relational expression for the effective modulus of fillers in the composite. The GPL by PMMA and CVD monolayer graphene satisfies the following equation:

$$E_{GPL} = E_{PMMA}(1 - V_{Gr}) + E_{Gr}V_{Gr} \tag{11}$$

where, E_{GPL}, E_{PMMA}, and E_{Gr} are the modulus of GPL, PMMA, and graphene, respectively, and V_{Gr} is the volume fraction of graphene. We identified the precise number of layers and composite thickness of graphene by rolled-up methods, and the formula can be changed as follows:

$$E_{GPL} = E_{PMMA}\frac{t_{PMMA}}{t_{PMMA} + t_{Gr}} + E_{Gr}\frac{t_{Gr}}{t_{PMMA} + t_{Gr}} \tag{12}$$

where, t_{PMMA} and t_{Gr} are the thickness of the PMMA matrix and graphene reinforcements, respectively.

Supplementary Fig. 6 shows the Young's modulus prediction of graphene calculated by the Halpin–Tsai model and the rule of mixtures. The aspect ratio was calculated at a = (100, 1,000, 10,000, and 1,000,000). When a >10,000, the Halpin–Tsai model approximates the rule of mixture, and at a = 1,000,000, the two plots overlap. When the aspect ratio of GPL exceeds 1,000,000, the rule of mixture was appropriately employed as a mechanical model to analyze our results.

3. <u>Mechanical reinforcement mechanism of the GPL</u>

From Figs. 3d–g of the main text, Pos G (ω_G) and Pos 2D (ω_{2D}) of graphene in the correlation Raman maps of T_g–GPL and GPL were scattered along the line with a slope of 2.2. We extracted the average values of the data points acquired from the S–GPL (0 % strained), T_g–GPL, and GPL's ω_{2D} of graphene from the Raman maps. The ω_{2D} values for S–GPL, T_g–GPL, and GPL were (2,687.74, 2,684.20, and 2,682.12) cm^{-1}, respectively. Based on Raman measurements with 514 nm excitations, the strain and ω_{2D} ratio of graphene supported by PMMA was observed to be −61.9 cm^{-1}/%[11, 12]. Accordingly, the residual tensile strain of heat-treated graphene for T_g–GPL and GPL is estimated to be (0.057 and 0.091) %, respectively.

These results have a lower value than the critical strain (0.4 %), where interfacial sliding of the PMMA–monolayer graphene interface occurs. Under the conformal contact and void-free conditions, interface sliding does not occur in a low-strain system, and the residual strain of graphene is dominant. Interestingly, the residual strain of graphene is strengthened in the hot-rolling mill process following stacking above the T_g. The T_g–GPL experienced bending during unfolding with thickness (t_c) and radius (r), where $\epsilon = t_c/2r$.

Subsequently, tensile strain is generated by hot-rolling mill process throughout the composite (including graphene). PMMA stretch is the main reason for strain generation by the hot-rolling mill process. By rolling mills, the area of PMMA increases and stretches as the thickness decreases. In a low-strain system with conformal contact, stretch is not limited to PMMA, but occurs throughout the GPM, including graphene. Figure 3b of the main text, and Supplementary Fig. 8, show the change in laminated graphene–PMMA composite morphology for the T_g–GPL and GPL. In the GPM rolled up with 1.2 cm diameter PTFT roller, the surface area increased by stretching, and consequently, the surface roughness decreased. CVD graphene has unavoidable wrinkle that occurs during the growth and transfer processes, which softens the modulus of graphene, and the morphology of stretched graphene refers to the flattening of the wrinkle[13, 14]. We previously calculated the graphene modulus of 1.09 TPa with a mechanical model. The large grain and high quality of graphene, as well as stretching, acted as an additional stiffness reinforce factor, and GPL has the maximum strengthening efficiency through graphene modulus close to intrinsic.

4. <u>*Thermal conductivity model of GPL*</u>

4.1. The parallel orientation model

The parallel thermal conductivity model for the layered structure is simply defined as follows:

$$K = V_f k_f + V_m k_m \tag{13}$$

where, K, k_f and k_m are the thermal conductivity of composite, filler, and matrix, respectively. Also, V_f and V_m are the volume fraction of filler and matrix. We applied the reported thermal conductivity value of CVD–graphene of (400, 600, and 2,500) W m^{-1} K^{-1} as a filler[15, 16].

4.2. The Maxwell–Eucken (ME) model

The ME model is based on continuous and dispersed phases[17, 18]. This model assumes a dispersed phase with small spheres in one continuous phase, and each sphere has a common dispersion state, separated from each other, to prevent distortion of the temperature distribution. The thermal conductivity of the composite by the ME model satisfies the following equation:

$$K = \frac{k_1 V_1 + k_2 V_2 \frac{3k_1}{2k_1 + k_2}}{V_1 + V_2 \frac{3k_1}{2k_1 + k_2}} \tag{14}$$

where, k_1 and k_2 are the thermal conductivity of continuous and dispersed phases (here, we used PMMA and graphene), and V_1 and V_2 are the volume fraction of each phase. Depending on the structure and dispersion state of the graphene or graphite flakes filler, the thermal conductivity of the composite can exceed the theoretical ME model[19]. Therefore, we calculated the ME model as the lower bound of randomly oriented structures.

Supplementary Figures

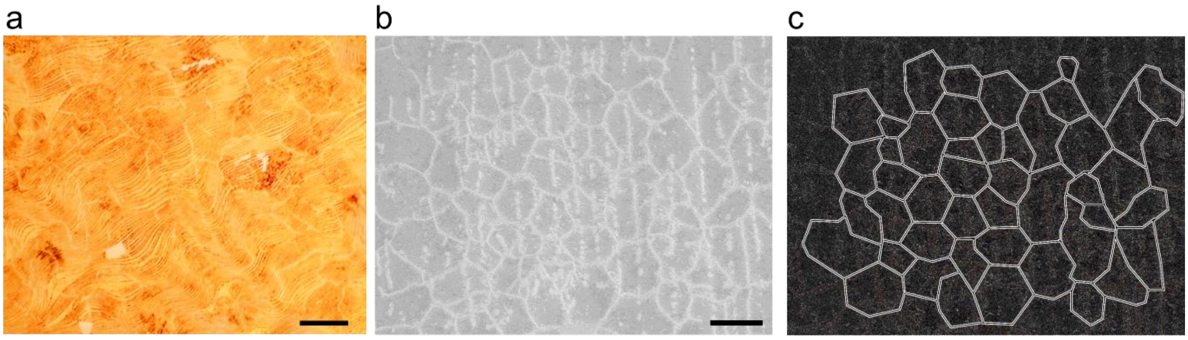

Supplementary Fig. 1 | Grain size analysis of the as-synthesized monolayer graphene. a, OM, and **b,** SEM images of graphene on the Cu foil (graphene/Cu) after the oxidation process. Graphene/Cu sample was baked for 2 h at 200 °C in air ambient condition. Scale bar: 20 µm. **c.** Grain boundaries were imaged from the original SEM images, in **b**. Image contrast is inverted for better visibility. The average grain size was calculated to be (14.18 ± 3.41) µm.

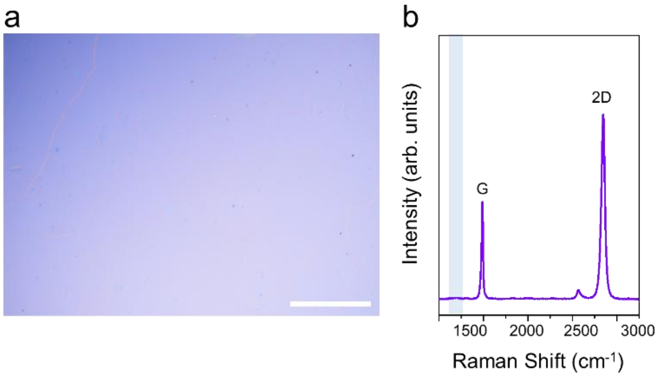

Supplementary Fig. 2 | OM images and Raman spectra of the prepared monolayer graphene. a, OM images of the transferred monolayer graphene on 300 nm SiO_2/Si. Scale bar: 100 µm. **b**, Raman spectra of the transferred monolayer graphene on 300 nm SiO_2/Si under 532 nm excitation. Source data are provided as a Source Data file.

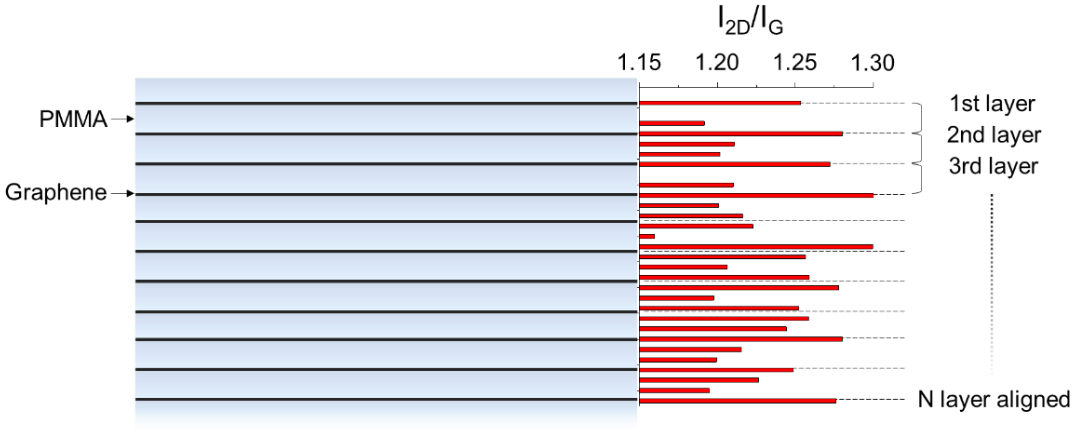

Supplementary Fig. 3 | Depth profiling of the GPL. For the depth profiling of Raman spectroscopy, the GPL was prepared under the determined PMMA (A4, 1,000 rpm) thickness condition. Raman intensity *vs.* depth trace measured in 100 nm steps is included in the graph. The intensity ratio of the 2D to G band (I_{2D}/I_G) by the graphene 2D peak was increased or decreased at equal intervals. A schematic of the Raman intensity by the alignment of the N-layers of graphene is at left. Source data are provided as a Source Data file.

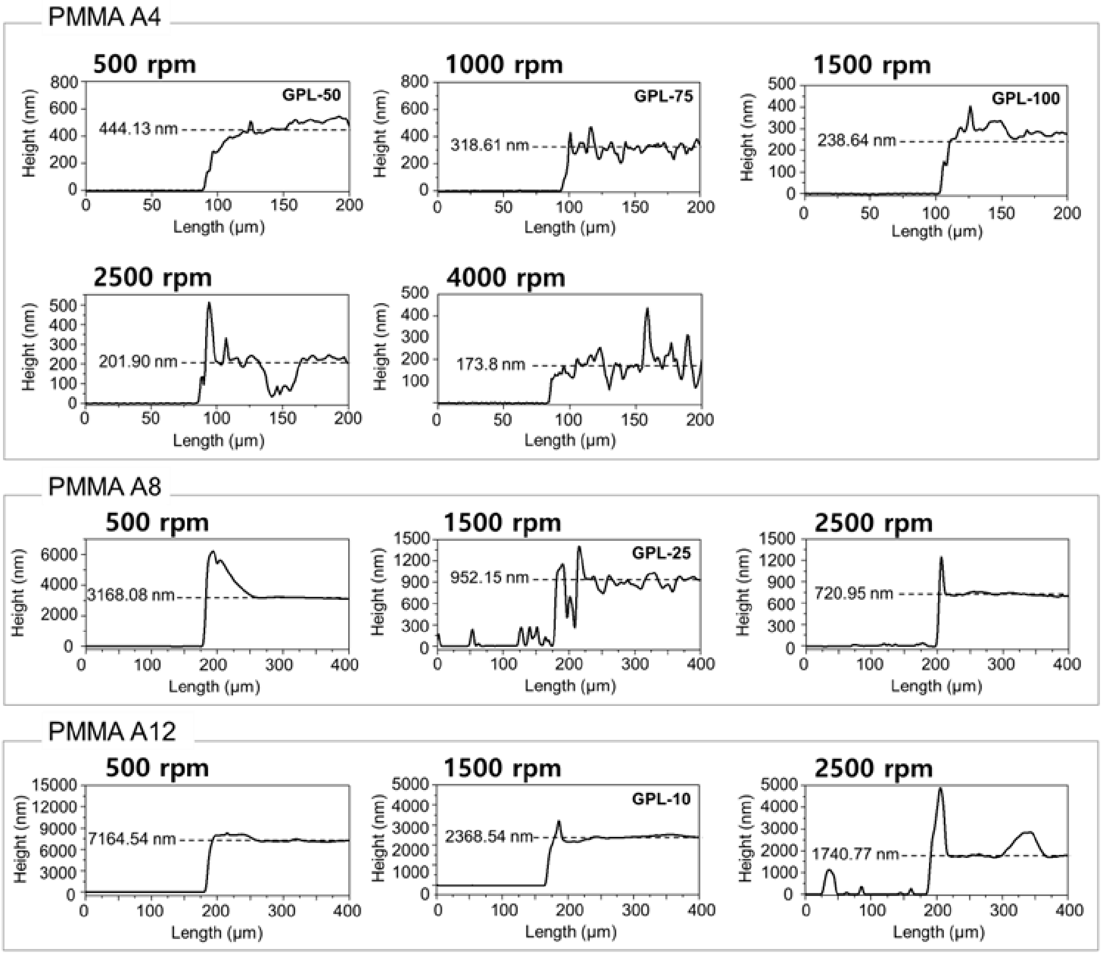

Supplementary Fig. 4 | Thickness analysis of the 1 layer (L)-GPM according to the concentration of PMMA solution and coating speed. After spin coating the PMMA on the graphene, the GPMs transferred onto the 300 nm SiO_2/Si. Thickness of the GPMs was measured by the Alpha-step. To manufacture the GPLs of the same thickness, we used different GPMs under the following conditions: GPL-10 (PMMA A12, 1500 rpm), GPL-25 (PMMA A8, 1500 rpm), GPL-50 (PMMA A4, 500 rpm), GPL-75 (PMMA A4, 1000 rpm), GPL-100 (PMMA A4, 1500 rpm). The graphene volume fractions for GPL-10, GPL-25, GPL-50, GPL-75, and GPL-100 are 0.019 vol.%, 0.046 vol.%, 0.098 vol.%, 0.142 vol.%, and 0.190 vol.%, respectively. Source data are provided as a Source Data file.

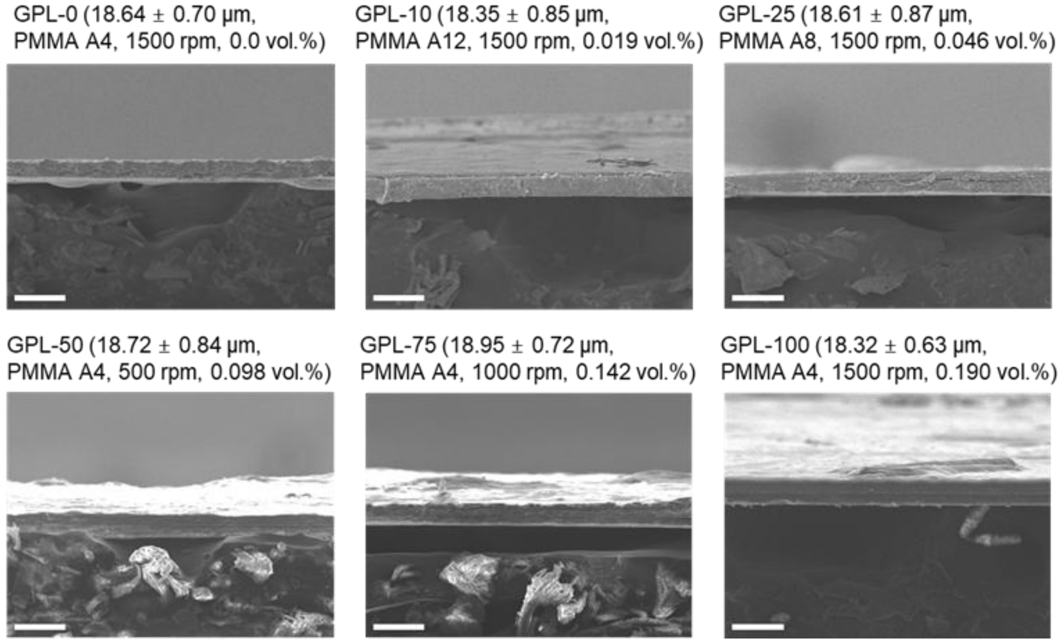

Supplementary Fig. 5 | Representative cross-sectional SEM images of GPLs. Average thickness of GPL-0 to GPL 100 was ((18.64 ± 0.70), (18.35 ± 0.85), (18.61 ± 0.87), (18.72 ± 0.84), (18.95 ± 0.72), and (18.32 ± 0.63)) μm, respectively. Scale bars: 50 μm, 47.6 μm, 50 μm, 35.6 μm, 35.6 μm, and 36.4 μm, respectively.

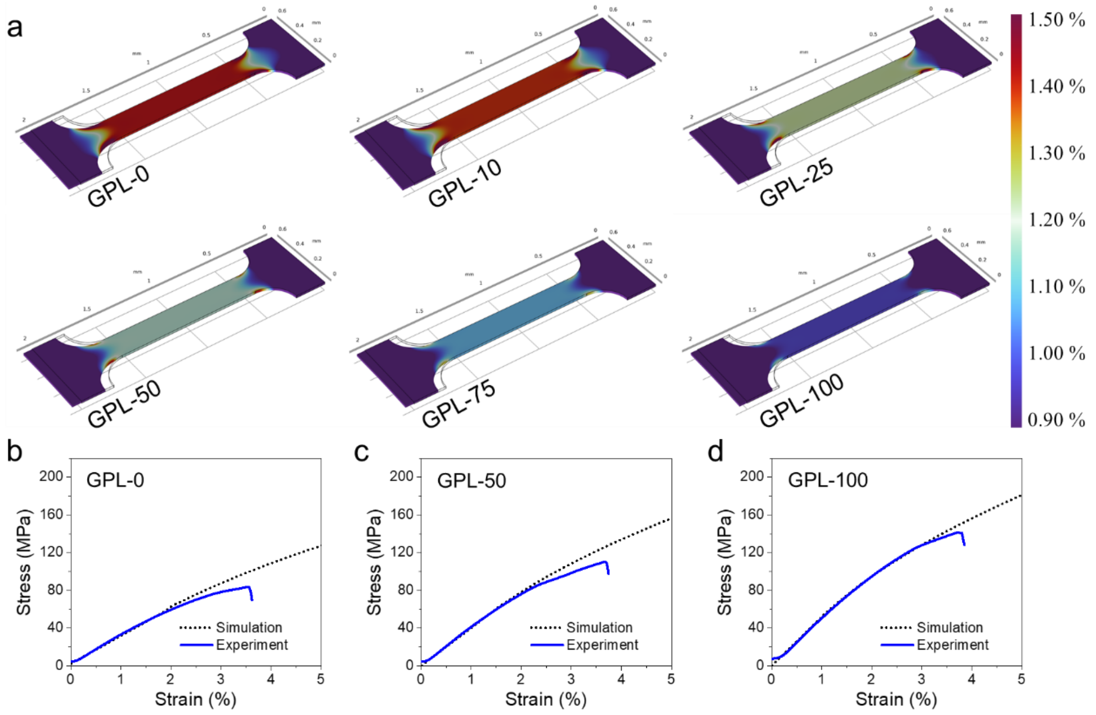

Supplementary Fig. 6 | FEM tensile simulations and stress–strain responses. a. Strain diagrams of graphene-PMMA structures corresponding to different graphene layer counts (0, 10, 25, 50, 75, 100 layers) at a stress level of 77.9 MPa. A distinct reduction in strain was observed as the number of graphene layers increases. **b-d.** Comparison between the experimental (blue lines) and simulated (dotted lines) stress-strain curves of GPL-0, GPL-50, and GPL-100. Source data are provided as a Source Data file.

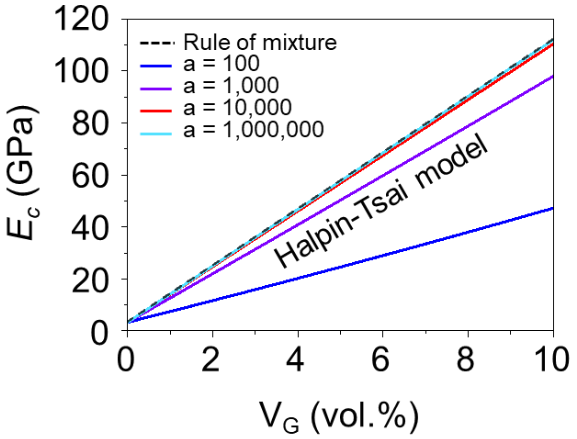

Supplementary Fig. 7 | Theoretical prediction of the elastic modulus of the GPL versus V_G. The solid line was predicted by the Halpin–Tsai model at a = (100, 1,000, 10,000, and 1,000,000), and the dashed line by rule of mixtures. Source data are provided as a Source Data file.

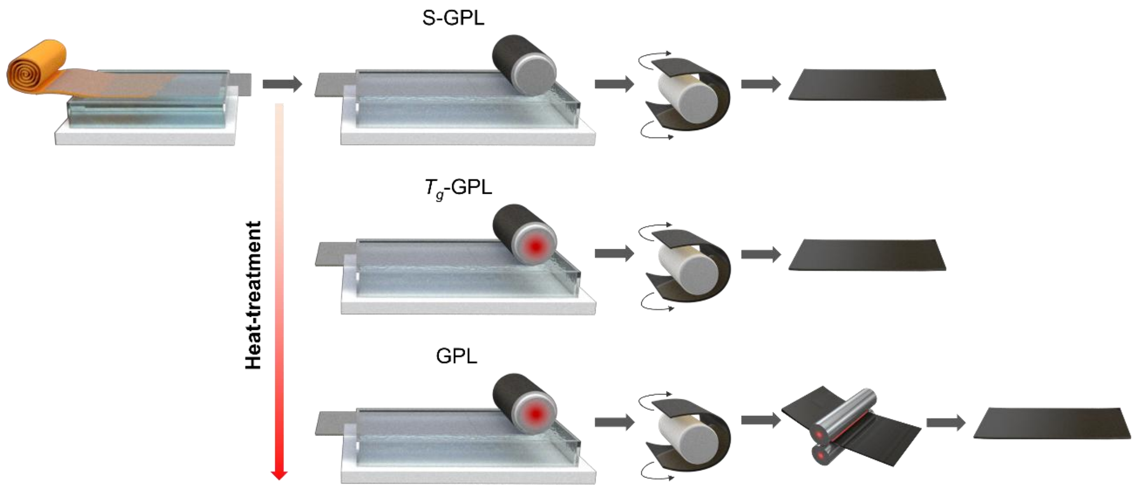

Supplementary Fig. 8 | Detailed manufacturing procedures of the GPL. Float–GPM is stacked by the rotation of the roller, and manufactured in three types. The GPM stacked at RT is called S–GPL. The T_g–GPL was manufactured by connecting a heater with a temperature above T_g-PMMA in the rolling-up process. The GPL shares the corresponding process as the T_g–GPL, and was manufactured by adding the hot-rolling mill process after unfolding.

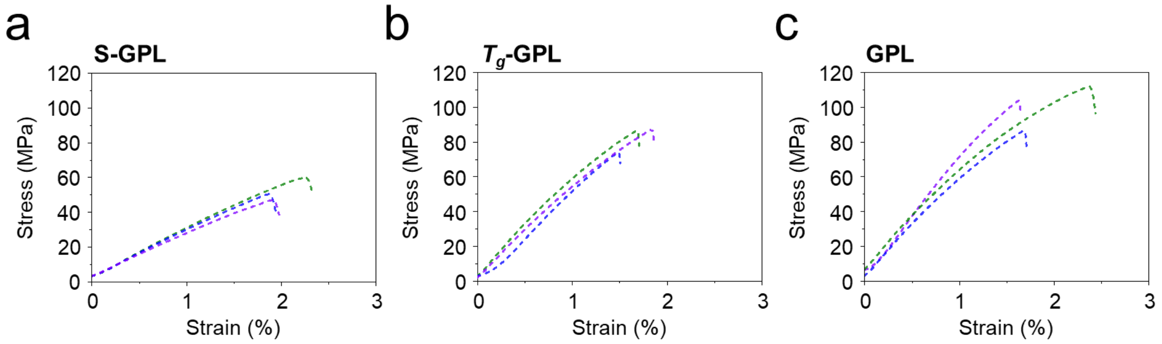

Supplementary Fig. 9 | Mechanical properties of the S–GPL, T_g–GPL, and GPLs. Strain–stress curve of **a**, S–GPL, **b**, T_g–GPL, and **c**, GPL. All samples were stacked with the identical GPM. Three specimens were measured for each sample. Source data are provided as a Source Data file.

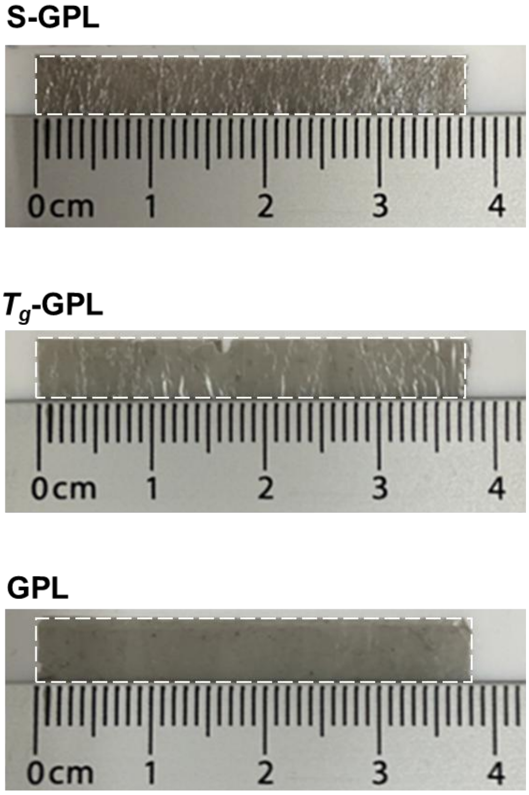

Supplementary Fig. 10 | Heat-treatment effect of the GPLs. Photograph of 3 different GPL samples (S–GPL, T_g–GPL, and GPL). Most of the wrinkles on the composite surface are diminished when stacked above the T_g, and after the hot-rolling mill process, were completely removed. A 1.2 cm PTFT cylinder produced the GPL with a length of 3.77 cm, but after the hot-rolling mill process, the GPL was slightly stretched.

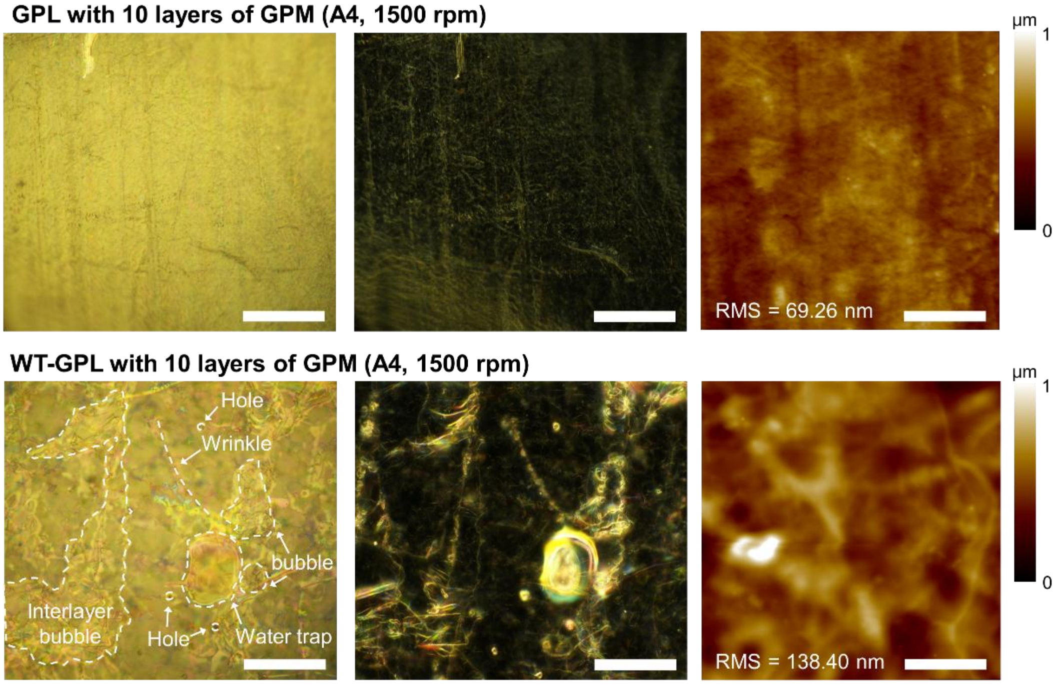

Supplementary Fig. 11 | Morphology analysis of GPL and wet transferred graphene-PMMA laminate. Representative high magnification (Left) bright field and (Right) dark field OM images of GPL and wet transferred graphene-PMMA laminate. In the corresponding AFM images, the root mean square of GPL is 69.26 nm and wet transferred graphene-PMMA laminate is 138.40 nm. Scale bar is 50 and 20 μm, respectively.

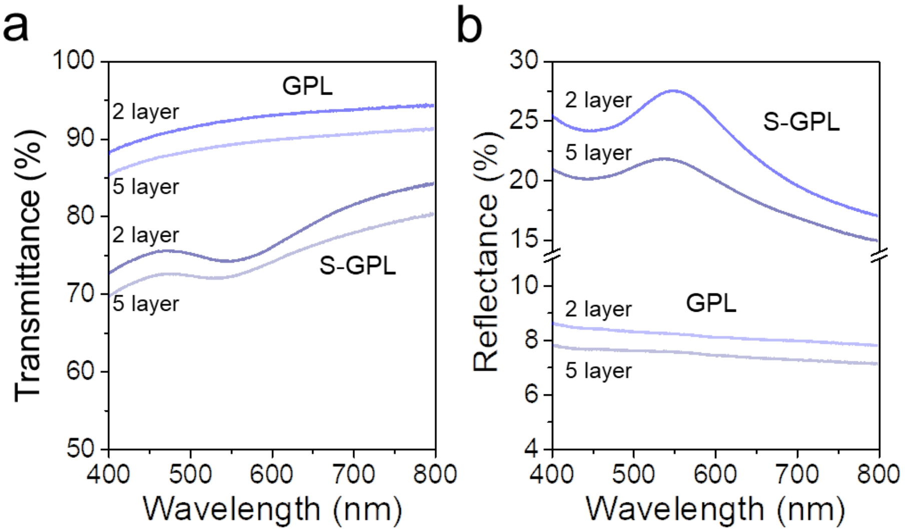

Supplementary Fig. 12 | Optical analysis of the S–GPL and GPL. a, Transmittance, and **b**, reflectance of the S–GPL and GPL with two and five layers of GPM. The transmittances of the S–GPL and GPL at 550 nm were (74.12 and 92.28) % in the 2 layers, and (72.13 and 89.17) % in the 5 layers, respectively. In the 2 and 5 layers, the reflectance of S–GPL was (27.50 and 21.67) %, and in the GPL was (8.22 and 7.55) %, respectively. Source data are provided as a Source Data file.

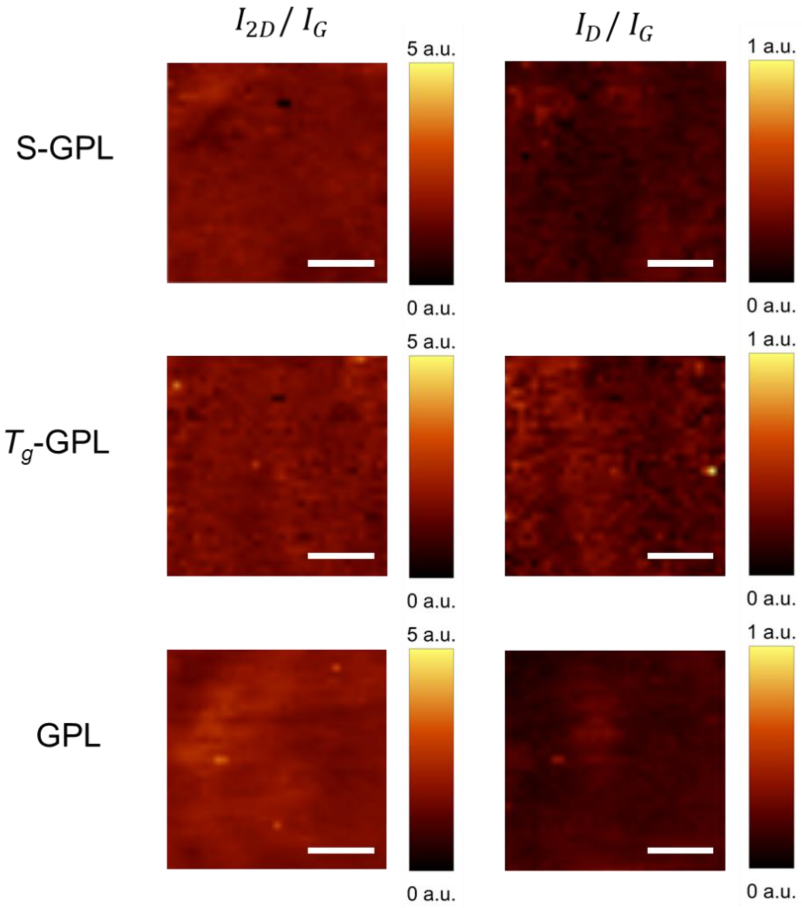

Supplementary Fig. 13 | Raman mapping of the S–GPL, T_g–GPL, and GPL. Raman intensity ratio of the 2D to G bands (I_{2D}/I_G) and D to G bands (I_D/I_G) was obtained from the S–GPL, T_g–GPL and GPL. Scale bar: 10 µm.

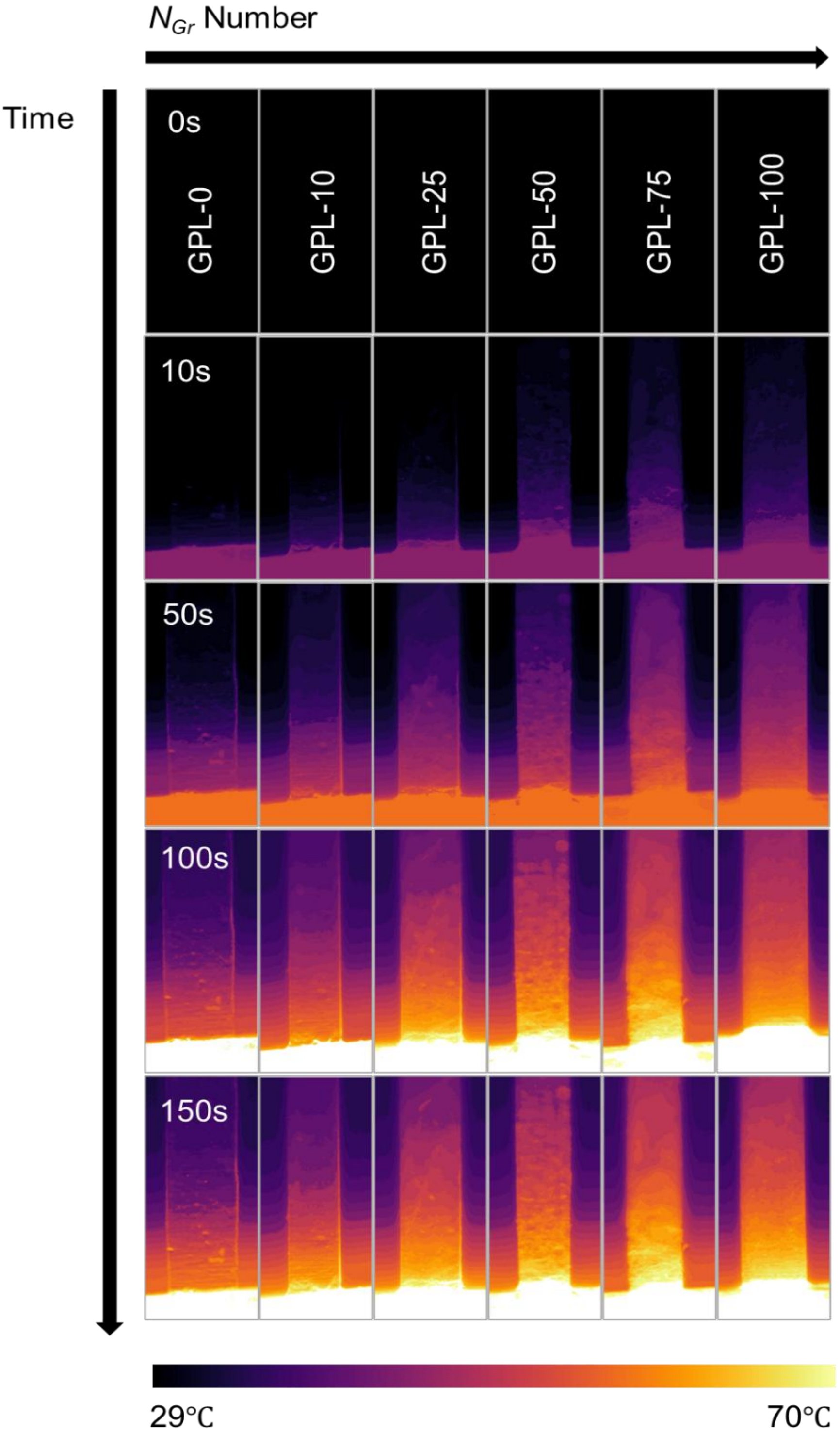

Supplementary Fig. 14 | Real-time visualization of heat conduction of the GPLs. Time-dependent heat transfer at (0, 10, 50, 100, and 150) s from GPL−0 to GPL−100 was observed through the IR camera. The GPLs with a width of 1 mm and a length of 10 mm show gradual heat transfer according to the number of graphene layers.

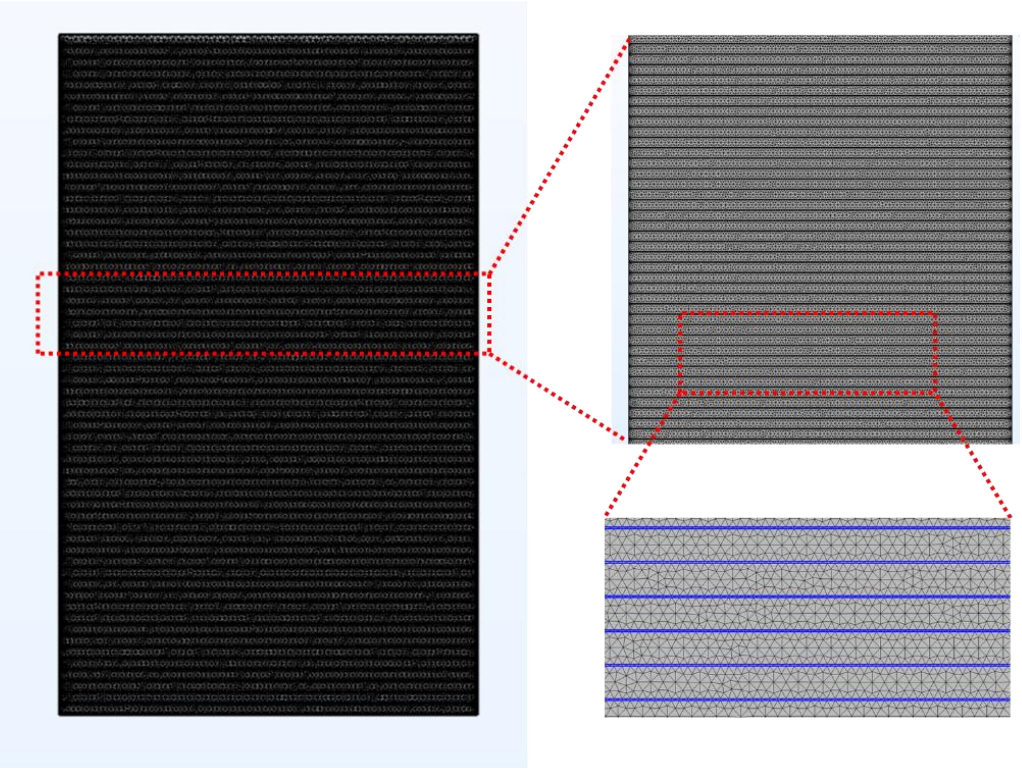

Supplementary Fig. 15 | Simulated graphene–PMMA laminate structure. The mesh size overview and zoom-in photo of mesh size in the PMMA structure (gray color) and graphene (blue color). According to the physics of the structure, the mesh size in the graphene areas is defined as "extremely coarse", while in the PMMA areas, is defined as "extra coarse".

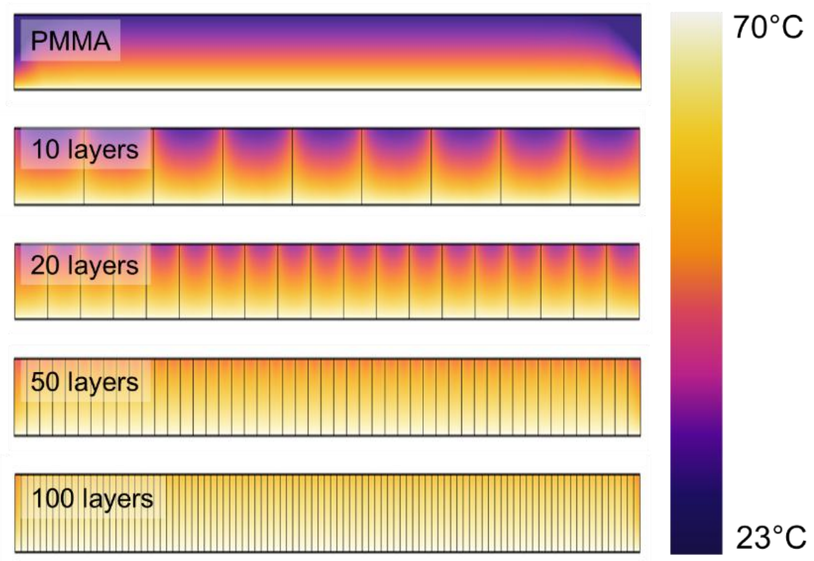

Supplementary Fig. 16 | Layer-dependent visualization of heat conduction of the graphene–PMMA laminate. Layer-dependent heat transfer of the graphene–PMMA laminate was simulated. There is a stronger correlation between the number of graphene layers and the heat transferring efficiency, as well as uniformity in temperature in the depth of the structure.

Supplementary Tables

Supplementary Table 1. Statistical thickness analysis of GPLs with different number of graphene layers.

		Thickness (μm)			Thickness (μm)			Thickness (μm)
**GPL-0**	#1	17.68	**GPL-10**	#1	18.04	**GPL-25**	#1	18.67
(0.0 vol.%)	#2	18.13	(0.019 vol.%)	#2	19.65	(0.046 vol.%)	#2	17.07
	#3	18.59		#3	17.41		#3	19.71
	#4	19.53		#4	19.14		#4	18.98
	#5	18.62		#5	17.93		#5	18.44
	#6	19.31		#6	17.92		#6	18.80
	**Average**	**18.64 ± 0.70**		**Average**	**18.35 ± 0.85**		**Average**	**18.61 ± 0.87**
		**Thickness (μm)**			**Thickness (μm)**			**Thickness (μm)**
**GPL-50**	#1	19.89	**GPL-75**	#1	19.59	**GPL-100**	#1	18.08
(0.098 vol.%)	#2	19.10	(0.142 vol.%)	#2	18.38	(0.190 vol.%)	#2	19.36
	#3	17.35		#3	19.57		#3	17.59
	#4	18.31		#4	19.52		#4	17.89
	#5	18.91		#5	18.71		#5	18.62
	#6	18.77		#6	17.91		#6	18.44
	**Average**	**18.72 ± 0.84**		**Average**	**18.95 ± 0.72**		**Average**	**18.32 ± 0.63**

Supplementary Table 2. Mechanical properties of the GPLs with different number of graphene layers.

		Tensile strength (MPa)	Modulus (GPa)	Fracture strain (%)
GPL-0	#1	83.65	3.26	3.54
	#2	79.69	3.23	3.30
	#3	75.46	3.50	2.73
	**Average**	**79.60 ± 4.10**	**3.33 ± 0.15**	**3.19 ± 0.42**
GPL-10	#1	79.09	3.22	3.58
	#2	90.01	3.54	3.35
	#3	82.87	3.45	3.27
	**Average**	**83.99 ± 5.54**	**3.40 ± 0.17**	**3.40 ± 0.16**
GPL-25	#1	97.91	3.83	3.59
	#2	105.01	4.17	3.13
	#3	91.06	3.76	2.99
	**Average**	**97.99 ± 6.98**	**3.92 ± 0.22**	**3.24 ± 0.31**
GPL-50	#1	110.50	4.31	3.42
	#2	110.35	4.28	3.67
	#3	112.47	4.12	4.00
	**Average**	**111.11 ± 1.18**	**4.24 ± 0.10**	**3.70 ± 0.29**
GPL-75	#1	114.24	4.63	3.65
	#2	127.43	4.91	3.67
	#3	122.25	4.55	4.26
	**Average**	**121.31 ± 6.65**	**4.70 ± 0.19**	**3.86 ± 0.35**
GPL-100	#1	138.10	5.12	4.05
	#2	141.09	5.27	3.7
	#3	144.87	5.71	3.58
	**Average**	**141.29 ± 3.29**	**5.37 ± 0.31**	**3.78 ± 0.24**

Supplementary Table 3. Mechanical properties of the GPL under three different conditions.

S-GPL

Sample number	Tensile strength (MPa)	Fracture strain (%)
#1	59.74	2.25
#2	50.27	1.87
#3	46.83	1.90
Average	52.28 ± 6.69	2.01 ± 0.21

T_g-GPL

Sample number	Tensile strength (MPa)	Fracture strain (%)
#1	85.77	1.66
#2	76.35	1.48
#3	87.22	1.84
Average	83.11 ± 5.90	1.66 ± 0.18

GPL

Sample number	Tensile strength (MPa)	Fracture strain (%)
#1	104.10	1.63
#2	86.56	1.69
#3	111.99	2.37
Average	100.88 ± 13.02	1.90 ± 0.41

Supplementary references

1. Packham, D. Work of adhesion: Contact angles and contact mechanics. *Int. J. Adhes. Adhes.* **16**, 121-128 (1996).

2. Zhang Newby, B.-M., Chaudhury, M. K. Friction in adhesion. *Langmuir* **14**, 4865-4872 (1998).

3. Andrews, E., Kinloch, A. J. Mechanics of adhesive failure. II. *Proc. R. Soc. Lond. A Math. Phys. Sci.* **332**, 401-414 (1973).

4. Schrader, M. E. Young-dupre revisited. *Langmuir* **11**, 3585-3589 (1995).

5. Tadmor, R. et al. Solid–liquid work of adhesion. *Langmuir* **33**, 3594-3600 (2017).

6. Prydatko, A. V., Belyaeva, L. A., Jiang, L., Lima, L., Schneider, G. F. Contact angle measurement of free-standing square-millimeter single-layer graphene. *Nat. Commun.* **9**, 1-7 (2018).

7. Selvakumar, N., Barshilia, H. C., Rajam, K. Effect of substrate roughness on the apparent surface free energy of sputter deposited superhydrophobic polytetrafluoroethylene coatings: A comparison of experimental data with different theoretical models. *J. Appl. Phys.* **108**, 013505 (2010).

8. Kozbial, A. et al. Study on the surface energy of graphene by contact angle measurements. *Langmuir* **30**, 8598-8606 (2014).

9. Ma, J. et al. Role of thin film adhesion on capillary peeling. *Nano Lett.* **21**, 9983-9989 (2021).

10. Affdl, J. C. H., Kardos, J. L. The Halpin-Tsai equations: A review. *Polym. Eng. Sci.* **16**, 344-352 (1976).

11. Anagnostopoulos, G. et al. Stress transfer mechanisms at the submicron level for graphene/polymer systems. *ACS Appl. Mater. Interfaces* **7**, 4216-4223 (2015).

12. Mohiuddin, T. et al. Uniaxial strain in graphene by raman spectroscopy: G peak splitting, grüneisen parameters, and sample orientation. *Phys. Rev. B* **79**, 205433 (2009).

13. Lin, Q.-Y. et al. Stretch-induced stiffness enhancement of graphene grown by chemical vapor deposition. *ACS Nano* **7**, 1171-1177 (2013).

14. Ruiz-Vargas, C. S. et al. Softened elastic response and unzipping in chemical vapor deposition graphene membranes. *Nano Lett.* **11**, 2259-2263 (2011).

15. Lee, W. et al. In-plane thermal conductivity of polycrystalline chemical vapor deposition graphene with controlled grain sizes. *Nano Lett.* **17**, 2361-2366 (2017).

16. Liu, J. et al. Thermal conductivity of giant mono-to few-layered CVD graphene supported on an organic substrate. *Nanoscale* **8**, 10298-10309 (2016).

17. Maxwell, J. C. *A Treatise on Electricity and Magnetism.* Vol. 1, Art. 202 (Clarendon Press, Oxford, 1873).

18. Eucken, A. Allgemeine gesetzmäßigkeiten für das wärmeleitvermögen verschiedener stoffarten und aggregatzustände. *Forsch. Geb. Ingenieurwes.* **11**, 6-20 (1940).

19. Tu, H., Ye, L. Thermal conductive PS/graphite composites. *Polym. Adv. Technol.* **20**, 21-27 (2009).
